# Oxidative Stress in Intracerebral Hemorrhage: Sources, Mechanisms, and Therapeutic Targets

**DOI:** 10.1155/2016/3215391

**Published:** 2015-12-30

**Authors:** Xin Hu, Chuanyuan Tao, Qi Gan, Jun Zheng, Hao Li, Chao You

**Affiliations:** Department of Neurosurgery, West China Hospital, Sichuan University, 37 Guoxue Alley, Chengdu, Sichuan 610041, China

## Abstract

Intracerebral hemorrhage (ICH) is associated with the highest mortality and morbidity despite only constituting approximately 10–15% of all strokes. Complex underlying mechanisms consisting of cytotoxic, excitotoxic, and inflammatory effects of intraparenchymal blood are responsible for its highly damaging effects. Oxidative stress (OS) also plays an important role in brain injury after ICH but attracts less attention than other factors. Increasing evidence has demonstrated that the metabolite axis of hemoglobin-heme-iron is the key contributor to oxidative brain damage after ICH, although other factors, such as neuroinflammation and prooxidases, are involved. This review will discuss the sources, possible molecular mechanisms, and potential therapeutic targets of OS in ICH.

## 1. Introduction

Intracerebral hemorrhage (ICH) remains a significant cause of morbidity and mortality throughout the world, although studies of ICH intervention have increased dramatically in the past decades [1]. Currently, there is no effective surgical or medical treatment available to improve the functional outcomes in patients with ICH because of its multiple injury mechanisms [1, 2]. Numerous preclinical studies show that secondary brain injury after ICH is caused by the interaction of cytotoxicity, excitotoxicity, oxidative stress (OS), and inflammation from the products of red blood cell lysis and plasma components [3, 4]. However, the precise pathophysiological mechanisms underlying ICH remain to be completely elucidated.

OS is a condition in which the overproduction of free radicals, mainly reactive oxygen species (ROS), exceeds the antioxidant capacity and subsequently leads to cell injury via directly oxidizing cellular protein, lipid, and DNA or participating in cell death signaling pathways [5]. OS has been implicated in neurodegenerative diseases of the central nervous system and stroke [6, 7]. There are three major types of ROS: the superoxide radical (O_2_^∙−^), the hydroxyl radical (^∙^OH), and hydrogen peroxide (H_2_O_2_) [8]. Reactive nitrogen species (RNS) are another major type of free radicals, which mainly consist of nitric oxide (NO) and its derivatives. NO is produced in neurons, endothelial cells, and activated astrocytes by nitric oxide synthase (NOS). Under physiological conditions, NO mediates neurotransmission and regulates neuronal survival, proliferation, and differentiation. Under pathological conditions, however, excessive NO can lead to OS via various mechanisms [9]. Moreover, NO reacts with O_2_^∙−^ to form the more toxic compound peroxynitrite (ONOO^−^), which can cause oxidation and nitration of tyrosine residues in proteins [10].

Experimental studies have confirmed that OS plays a pivotal role in cerebral injury following ICH. The oxidative products of macromolecules significantly increased, whereas antioxidant enzymes, such as superoxide dismutase (SOD), glutathione peroxidase (GPx), and catalase, correspondingly decreased as a result of ICH [11, 12]. Free radical scavengers proved to be effective in neuroprotection in animal ICH models [13, 14]. Moreover, oxidative markers, leukocyte 8-hydroxy-2′-deoxyguanosine and lipid hydroperoxides, are detected in association with long-term and short-term clinical outcomes, respectively, after spontaneous ICH [15, 16]. However, no antioxidant has been applied in patients with ICH because of the unclear mechanism of OS-related injury. The current review attempted to illustrate the knowledge regarding ICH-related OS and its possible molecular mechanism and to discuss the potential targets of intervention for future research.

## 2. Primary and Secondary Brain Injury after ICH

Primary brain injury is caused by immediate physical disruption to the neurovascular architecture surrounding the hemorrhagic site due to sheering force and the mass effect of an ICH. Surgical clot evacuation targeting the primary injury has failed due to the extra adverse effects of the surgical procedure [17, 18]. Many clinical trials on minimally invasive surgery for ICH evacuation have thus been performed with potentially improved functional outcomes [19, 20]. However, there are several drawbacks, such as the long time required for an adequate blood evacuation, a relatively high risk of infection and rehemorrhage, and intensive labor and resource consumption [21]. After the onset of bleeding, hematoma enlargement further exacerbates brain damage in 20–40% patients within 24 hours [22]. Hypertension may be a modified factor affecting hematoma growth [23].

When initial bleeding stops and a stable hematoma is formed, a cascade of events occurs to induce secondary brain injury. Thrombin is instantly produced after ICH to stop bleeding, but it also contributes to early neural and endothelial injury [24]. Inflammatory cells infiltrate and damage perihematoma viable brain tissue by excreting a variety of cytokines and chemokines [25]. Another contributor to brain injury after ICH is hemoglobin and its metabolite released via erythrocyte lysis in hematoma [26]. As the major component of hemoglobin, heme can be degraded into iron, carbon monoxide, and biliverdin by heme oxygenase (HO). Iron overload in the brain after hemorrhage subsequently generates abundant ROS, resulting in neurotoxicity [27]. These overlapped mechanisms interact and result in blood brain-barrier (BBB) disruption, neuronal loss, and gliosis with permanent neurological deficits.

## 3. OS in ICH

ROS are byproducts of cellular metabolism and are mainly generated by mitochondria in living cells [28]. They are highly active with a short half-life, making them very difficult to analyze directly in the laboratory [29]. OS is usually assessed by indirectly measuring the oxidized products of macromolecules. 8-Hydroxy-2-deoxyguanosine (8-OHdG) is a widely used biomarker of in vivo oxidative DNA damage. Both malondialdehyde (MDA) and 4-hydroxy-trans-2-nonenal (HNE) are lipid peroxides produced by free radical attack. Dinitrophenyl (DNP) and protein carbonyl can be measured to quantify protein oxidative damage. The detection of oxidized hydroethidine (HEt) is specifically used to assess O_2_^∙−^ production in vivo because HEt can pass through the BBB and be selectively taken up by neuron and glia cells and oxidized by O_2_^∙−^ to ethidine (Et), which provides a red fluorescence signal [30].

In a rodent ICH model, 8-OHdG and DNP increased along the same time course, with peak production at 3 days after ICH, suggesting the presence of OS in ICH [12]. Additionally, the level of MDA increases and is correlated with apoptosis following ICH, indicating that OS contributes to ICH-induced brain injury [31]. Moreover, brain white matter is also damaged as a result of protein oxidation in a porcine ICH model [32].

Recently, OS was reported to exert a prognostic effect in ICH patients. A prospective study analyzing blood samples from 64 ICH patients revealed that elevated level of leukocyte 8-OHdG was associated with lower 30-day Barthel Index independent of traditional prognostic factors [15]. Another prognostic study reported that the serum lipid hydroperoxide (ROOH) concentration was a predictor of poor clinical outcome in ICH survivors and was positively correlated with short-term mortality [16]. In contrast, Mantle et al. observed similar levels of protein carbonyl and antioxidants in ICH and control cases, suggesting that there may be no increased oxidative damage in ICH [33]. This unexpected result is questionable because the tissue (peritumor or aneurysm tissue) used as a control for the oxidative measurement may be pathologically compromised with potentially elevated levels of OS and therefore is not a qualified control [34].

## 4. Free Radical Sources after ICH

### 4.1. Mitochondria Dysfunction

Physiologically, 1–3% of all electrons in the electron transport chain in mitochondria leak, generating superoxide radicals, that can be neutralized by normal antioxidant systems [28]. During ICH, mitochondria dysfunction occurs, and substantial ROS production follows [35, 36]. Kim-Han et al. detected an obvious reduction in the oxygen consumption rates of mitochondria in ICH patients, indicating that mitochondria dysfunction, and not ischemia, is responsible for the decreased oxygen metabolites after ICH [35]. Direct evidence of ROS from malfunctioning mitochondria was reported in a recent study, which found that a mitochondrial ROS-specific scavenger can significantly alleviate the increased ROS following ICH [37]. The mechanism of excessive ROS formation by mitochondria after ICH remains unclear but may be partially attributable to mitochondrial permeability transition pore (MPTP) because the inhibition of MPTP can attenuate ROS production [37].

### 4.2. Hb-Heme-Iron

As the most abundant erythrocyte protein, hemoglobin (Hb) is released into the extracellular space via complement-mediated cell lysis in the hours after ICH and is a potent mediator of OS-induced injury [38, 39]. Both in vitro and in vivo investigations have shown that ROS is highly produced after exposing Hb to cell culture or injecting Hb into mouse striatum [39–41]. Katsu et al. studied the temporal change of ROS in a Hb-injection rat model and observed remarkable ROS production as early as 1 h, which increased at 24 h [42]. Recently, NO, a form of RNS, has also been found to be overproduced because of NOS activation and leads to BBB disruption after infusing Hb into rat brain [43]. Regarding the prooxidant mechanism of Hb, it is commonly believed that iron released from its degradation is responsible for oxidative damage because an iron chelator may block Hb-induced neurotoxicity [44]. In fact, Hb itself can release a large amount of superoxide during spontaneous, nonenzymatic oxidation to oxyhemoglobin and methemoglobin [45, 46].

Heme, released from methemoglobin, quickly oxidizes to form hemin, which also triggers oxidative damage in brain tissue around the hematoma. An in vitro experiment demonstrated that hemin exposure leads to cell death, preceded by a significant, iron-dependent increase in ROS [47]. Nevertheless, another in vitro study showed that hemin could stimulate lipid peroxidation, irrespective of iron mediation, because the reaction could not be inhibited by deferoxamine or transferrin [48]. Hence, the mechanism of hemin-related oxidative damage partly involves its breakdown to iron by HO, similar to that of Hb [49]. Indeed, hemin is redox-active and can react with peroxides to produce cytotoxic free radicals [48, 50]. Moreover, hemin can intercalate into the cell plasma membrane, facilitating lipid peroxidation [51]. Given the effect of hemin in preclinical studies, biphasic functions are observed. Hemin-induced brain injury is evidenced by increased brain water content at 24 hours after intracerebral hemin infusion [46]. In contrast, systemic hemin treatment is neuroprotective after ICH [52]. Although the mechanisms underlying the protection provided by systemic hemin administration are poorly understood, it is clear that most hemin is in circulation rather than in the brain.

Iron overload is involved in secondary brain injury, leading to neuronal death, brain edema, and neurodeficits after ICH [53, 54]. Intracerebral iron overload begins within 24 h, peaks at 7 days, and continues for at least a month after hemorrhage [55]. Excessive iron in the extracellular space induces oxidative damage via the Fenton reaction, which yields ROS, especially toxic hydroxyl radicals [56]. Direct evidence of iron-mediated oxidative injury has shown that injecting FeCl_2_ into rat brain causes oxidative DNA damage [11, 12]. The strongest finding supporting the hypothesis of iron-mediated oxidative brain injury is that iron chelators decrease iron accumulation, attenuate ROS generation, exert anti-inflammatory effects, and improve neurological function [57, 58].

### 4.3. Inflammatory Cells

Neuroinflammation is recognized as a vital factor in the pathophysiology of ICH-induced brain injury and is characterized by microglia activation, leukocyte infiltration, and cytokine and chemokine production [3, 4, 25]. In addition to the release of inflammatory factors, the activation of inflammatory cells following ICH, initially to remove oxidative toxins, also participates in ROS production.

As one type of innate immune cell within the brain, microglia are rapidly activated within 1 h after ICH, peaking at 3–7 days and persisting for several weeks [59]. Hb is a powerful activator of microglia via toll-like receptors [60]. The imbalance of the phenotypic shift between the M1 and M2 phenotypes of microglia contributes to a large release of ROS in addition to proinflammatory factors [61]. Cell experiments have shown that microglia can induce ROS production in vitro [58, 59]. Furthermore, the inhibition of microglia was reported to decrease the ROS production and brain damage volume in an ICH animal model [62].

Neutrophils are the earliest leucocytes to enter the brain after ICH. The role of neutrophils in radical production during ischemic brain stroke has been confirmed by reduced radical formation after neutrophil depletion [63]. OS-related brain injury is part of the pathogenesis mechanism of neutrophil infiltration after ICH [64]. The inflammation linked to OS following ICH indicates that neuroinflammation and OS are intercalated in ICH-induced secondary brain injury.

## 5. Prooxidase in ICH

The process of OS is related to the activation of many prooxidases in many diseases. The prooxidases that are reported in ischemia stroke include NADPH oxidase (NOX), cyclooxygenase (COX), xanthine oxidase, and nitric oxide synthase [NOS] [65]. In ICH, NOX and NOS are most commonly studied [10, 66–68].

### 5.1. NADPH Oxidase

NOX is a major source of ROS and is mainly composed of five subunits: a large gp91^phox^ and a smaller gp22^phox^ subunit in the plasma membrane and p47^phox^, p67^phox^, and p40^phox^ subunits in cytoplasm [65]. Once cytosolic p47^phox^ is phosphorylated upon stimulation, it binds to the components of the plasma membrane and activates NOX, which can transfer electrons from NADPH to oxygen, forming superoxide [66]. Seven NOX isoforms, NOX1 to NOX5 and Dual Oxidases 1 and 2, have been identified among which NOX2 (gp91^phox^) is abundant in the brain [67–69]. Tang et al. found that the OS resulting from activation of NOX2 contributes to the severity of ICH and promotes brain injury by comparing wild-type and gp91^phox^ knockout mice [67]. The gp91^phox^ knockout hemorrhagic mice showed lower levels of oxidative product, ICH volume, brain water content, neurological deficit, and mortality rate [67].

Another study by Zia and colleagues showed that the induction of NOX2 could cause OS and worsen brain injury, whereas the inhibition of NOX2 by apocynin suppresses ROS production and confers neuroprotection in rabbit pups with germinal matrix hemorrhage-intraventricular hemorrhage (GMH-IVH) [68]. Moreover, OS resulting from NOX2 activation not only deteriorated ICH-related injury but was associated with the occurrence of ICH in hypertensive mice [70]. However, the same NOX2 inhibitor that exerts a protective property in the GMH-ICH model by preventing p47^phox^ subunit translocation exhibits no effects on enhanced NOX2 activity, lipid peroxidation, brain edema, or neurological dysfunction in a rat ICH model [71]. It is possible that different species (rabbit versus rat), hemorrhagic locations (GMH-IVH versus basal ganglia hemorrhage), and bleeding (autologous artery blood versus collagenase) are responsible for these opposing conclusions.

### 5.2. Nitric Oxide Synthase

There are three isoforms of NOS accounting for NO production: neuronal NOS (nNOS), endothelial NOS (eNOS), and inducible NOS (iNOS). The first two are constitutively expressed, and their activities are calcium dependent, whereas the last one is synthesized by the induction of proinflammatory cytokines, independent of calcium regulation [72].

The activation of NOS after ICH has been demonstrated in many studies. Using the autologous blood model, Zhao et al. reported the temporal profile of iNOS and nuclear factor-*κ*B (NF-*κ*B) and found that the maximal detection of iNOS paralleled the peak concentration of NF-*κ*B at 3 days after ICH, suggesting that iNOS may be mediated by NF-*κ*B because the downstream gene products of NF-*κ*B include iNOS [73]. Other investigators detected NOS overexpression and suggested the role of the NOS/NO/ONOO^−^ pathway in BBB disruption using the Hb-injection rat model [10, 43]. In contrast, administering nNOS inhibitor after ICH was found to protect BBB integrity and decrease both neuronal death and neurological deficits [74]. Moreover, iNOS knockout mice present significantly less brain edema after collagenase-induced ICH [75]. Therefore, NOS might be a therapeutic target.

The molecular mechanisms for NOS activation after ICH are primarily NF-*κ*B dependent [9, 73]. Thrombin and proinflammatory cytokines, such as TNF-*α* and IL-1, can induce iNOS expression in microglia via the PKC/p38MAFP/NF-*κ*B pathway [76]. Hemin also activates the NF-*κ*B transcription factor via an undefined mechanism [77, 78]. In addition, high levels of glutamate activate NOS through the NMDA receptor with subsequent Ca^2+^ influx by phosphorylating IKB and NF-*κ*B translocation [74, 79].

## 6. Antioxidative System in ICH

### 6.1. Heme Oxygenase

Extracellular heme binds to hemopexin to enter neuronal cells through the hemopexin receptor or heme carrier protein 1 [80]. Intracellular heme is then degraded into iron, carbon monoxide, and biliverdin. HO is the rate-limiting enzyme for this catabolic process with two active isoenzymes: the inducible HO-1 and the constitutively active HO-2. HO-1 is barely detected in the brain under normal conditions but is induced in microglia/macrophages after ICH, whereas HO-2 is normally expressed in neurons, accounting for the vast majority of HO activity in the brain [81].

The antioxidant effects of these enzymes on ICH-induced secondary brain injury are debatable and have been thoroughly reviewed by Chen-Roetling et al. [82]. Their roles are variable, depending on the different ICH models used and various cellular types affected [82]. Compared to wild-type mice, HO-2 knockout was found to attenuate brain injury, remarkably reducing cell loss, striatal protein, and lipid oxidation in a blood-injection model, but worsened the outcome by increasing perihematomal lesion volume, neuroinflammation, and edema in a collagenase-injection model [83, 84]. Conversely, HO-1 knockout exerted a beneficial effect on outcome in a collagenase-induced ICH model [85]. These disparate conclusions are partly explained by the diverse injury mechanisms between the blood- and collagenase-injection ICH models and the different distributions and expression timing of HO-1 and HO-2 [82].

### 6.2. Superoxide Dismutase

SOD is a key antioxidant enzyme that can detoxify O_2_^∙−^ to H_2_O_2_, which is further converted to H_2_O by catalase or GPx. According to the specific cellular distribution and metal cofactors, SOD can be categorized into copper/zinc SOD (SOD1) in the cytosol and manganese SOD (SOD2) in the mitochondria and extracellular SOD (SOD3) [86].

Experimental animal studies have shown that free radical scavenging systems are destroyed after ICH. More specifically, evidence suggests that the levels of SOD1 and SOD2 decrease as the ROS level increases 1 day after lysed erythrocyte infusion in rats [87]. Chen et al. confirmed the damaged antioxidant system with elevated lipid oxidation and decreased SOD activity 1 day after ICH in the ventricle [88]. Clinically, decreased plasma SOD and reduced total superoxide scavenger activities have been observed in ICH patients within 1 day after onset [89]. However, SOD1 was found to increase from 1 day after ICH induced by whole blood infusion and peak at 7 days in one study [90]. These contrasting results require further investigation.

Given the protective effect of SOD1, exogenous or endogenous enhancement of SOD1 has been attempted to alleviate the oxidative damage in ICH. SOD1 overexpression in transgenic rats was linked to reduced OS, BBB disruption, and neuronal apoptosis in a Hb-injection model [42]. A recent study on cell replacement therapy in ICH found that neural stem cells (NSCs) overexpressing SOD1 3 days after ICH could increase neuronal survival, indicating that SOD1 enhancement alone or combined with other treatments may be effective in ICH [91]. Moreover, SOD1 hyperexpression is also protective against the spontaneous occurrence of ICH in hypertensive mice by decreasing superoxide. Fewer occurrences, smaller size, and a lower number of ICH are observed in SOD1 transgenic mice than those in SOD1-deficient mice [92]. However, chemically synthesized SOD with extended half-life and improved BBB permeability was reported to have no effect in a collagenase-induced ICH model when intravenously administered [93]. This failure may be partially ascribed to the insufficient dosage used [93].

### 6.3. Nuclear Factor Erythroid-2 Related Factor 2 (Nrf2)

Nrf2 is a basic region-leucine zipper protein that controls the genomic regulator of the cellular antioxidant defense system, including the HO and SOD mentioned above [94]. ROS can activate the Keap1/Nrf2/ARE pathway to counteract oxidative damage after ICH as an adaptive response [90, 95, 96]. Keap1 is an OS sensor and negatively regulates Nrf2. Once exposed to ROS, Nrf2 dissociates from Keap1, translocates to the nucleus, and activates antioxidant response element (ARE) dependent cytoprotective genes that mediate cell survival [97]. Nrf2 increases significantly from 22 h and peaks at 8 h, whereas Keap1 shows a corresponding decrease in the perihematoma region in ICH rats [90]. These opposing expression changes suggest that Nrf2 is activated by Keap1 suppression after ICH. Moreover, the neuroprotection of Nrf2 indicates that Nrf2 knockout mice suffer more brain damage associated with the increased production of ROS and apoptosis [95, 96] and that Nrf2 activation could reduce peroxide formation by augmenting the antioxidative capacity and hematoma clearance after ICH [98]. Hence, Nrf2 activation by pharmaceutical drugs is a promising target to attenuate OS-induced brain injury following ICH.

Recently, dimethyl fumarate (DMF), a fumaric acid ester that has been approved by the FDA as a treatment for patients with relapsing-remitting multiple sclerosis (MS) [99], demonstrated a beneficial effect by activating Nrf2 in rodent ICH models [100, 101]. In the study by Zhao et al., rats and mice, including Nrf2 knockouts, were initially subjected to intracerebral injection of blood and were then treated with DMF at a clinically relevant dose [100]. The results showed that treatment with DMF activated Nrf2, induced antioxidative enzymes, reduced brain edema, and ultimately enhanced neurological function. Additionally, enhanced hematoma resolution was observed in in vitro experiments by evaluating the phagocytic functions of primary microglia in culture. Iniaghe and colleagues found that upstream casein kinase 2 promoted Nrf2 translocation to exert a neuroprotective effect after DMF treatment [101]. These findings are important. Because DMF is currently approved for clinical use for MS, clinical translation will be relatively easy once the efficacy of DMF on ICH is confirmed in a clinical trial.

## 7. OS and Death Signaling Pathways in ICH

Numerous brain stroke studies have revealed that ROS/RNS not only directly oxidize cellular macromolecules, such as lipids, proteins, and nucleic acids, associated with oxidative damage, but also are involved in the death signaling pathways. The molecular mechanisms of ROS-mediated cell death in brain ischemia have been thoroughly studied and reviewed elsewhere [29, 65]. Briefly, there are three major OS-mediated pathway activations, including the PI3K/Akt, MAPK/P38, and NK-*κ*B pathways [29]. Cytochrome c-mediated apoptosis is another critical pathway that is mitochondria dependent [102]. These OS-induced death signaling pathways have also been discussed in subarachnoid hemorrhage [103].

Free radicals can induce apoptosis, and antioxidant therapy can reduce neuronal apoptosis after ICH [104, 105]. Few studies have focused on the precise mechanism of ROS/RNS-induced apoptosis or necrosis in the setting of ICH. In vitro Hb oxidative neurotoxicity was attenuated by inhibitors of protein kinase C (PKC) and protein kinase CK2, suggesting that the PKC/CK2 pathway might participate in Hb-induced apoptosis, independent of HO activity [106]. However, the ERK pathway is involved in heme-mediated neuronal death by affecting HO-1 activity [107, 108]. The NF-*κ*B pathway has also been detected in mediating Hb-induced apoptosis [108]. Moreover, the JNK pathway was reported to be activated following iron infusion, and the inhibition of JNK activation reduces apoptotic neuronal cell death and improves functional outcome [109, 110]. Other studies have shown that caspase cascades are activated by OS after hemoglobin explosion in primary neuronal cultures [36] and that ROS-induced apoptosis is related to cytochrome c release in the ICH model [95].

**Figure 1:**
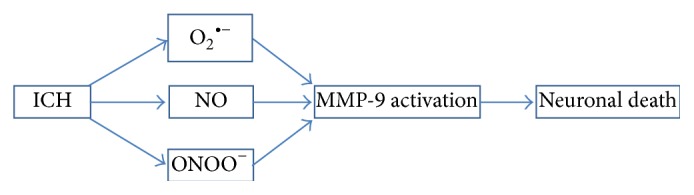
The OS-induced death pathway mediated by MMP-9. Hb released into extracellular space via complement-mediated cell lysis after ICH is a potent oxidant which can produce a plenty of free radicals such as superoxide (O_2_^∙−^), NO, and their conjunctive metabolite, peroxynitrite (ONOO^−^). These ROS/RNS activate MMP-9 possibly through NF-*κ*B activation and finally lead to neuronal death. ICH: intracerebral hemorrhage; MMP-9: matrix metalloproteinases-9.

Although the ROS-mediated apoptotic signal pathway after ICH remains unclear, recent findings have shown that MMP-9 is an important mediator linking ROS/RNS with cell death following ICH [42, 95, 111, 112]. MMP-9 has been reported to elevate early, with a peak at 2-3 days, and is associated with apoptosis in the acute phase of ICH [113, 114]. The MMP inhibitor, GM6001, ameliorated neuronal death when administered within 72 h in a mouse ICH model [113]. Both in vitro and in vivo experiments have shown that Hb-induced ROS contributes to MMP-9 activation [42, 111]. NO derived from iNOS has also been reported to directly activate MMP-9 [112]. Moreover, a recent study by Ding et al. demonstrated that superoxide, NO, and their potent toxic metabolite peroxynitrite (ONOO^−^) participate in the activation of MMP-9 via the following two mechanisms [115]. First, ONOO^−^ directly modifies pro-MMP through S-nitrosylation and then activates MMP-9. Second, NF-*κ*B is indirectly upregulating and mediates the transcription of MMP-9 [116]. The strong evidence supporting MMP-9-mediated OS-induced cell death is based on the fact that scavenging or decomposing ROS/RNS significantly decreases MMP-9 activity and subsequent neuronal death. SOD1 overexpression or free radical scavenger U83836E successfully reduced OS, MMP-9 levels, and subsequent apoptosis after intrastriatal Hb injection [42, 111]. iNOS inhibition by osteopontin to prevent NO production also suppressed MMP-9 activation and rescued neuronal cells in the perihematoma region in a mouse collagenase-induced mouse ICH model [112]. Additionally, FeTPPS, a type of ONOO^−^ decomposition catalyst, decreased the levels of ONOO^−^ and MMP-9 activity, followed by reduced apoptosis, in a Hb-injection rat model [115]. Therefore, ROS/RNS and MMP-9 may constitute a crucial cell death pathway in ICH (Figure 1).

## 8. Therapeutic Targets and Clinical Trial

Given the abovementioned multiple sources of ROS generation and injured oxidant scavenger systems during OS-induced damage in ICH, several potentially therapeutic targets are discussed.

### 8.1. Blocking the Sources of ROS Production

Because intraparenchymal blood is the origin of many prooxidant toxins, including Hb, heme, and iron, it is reasonable to suppose that blood evacuation may reduce oxidative damage if the surgery results in no or minimal additional new injury. Hence, minimally invasive surgery (MIS) for clot evacuation may represent a therapeutic strategy for the prevention of secondary oxidative damage. Animal studies have demonstrated that MIS alone or combined with other therapy can improve neurofunction with decreased oxidative injury and reduced apoptosis [117, 118]. More recently, clinical trials with small sample sizes investigating newly applied mechanical devices have reported promising outcomes [119, 120]. A multicenter, randomized, controlled study by our center comparing MIS with routine craniotomy is ongoing and involves 2448 ICH patients [121]. The clinical results will provide valuable information regarding the effect of MIS on the prognosis of patients with ICH.

Strategies targeting chelating individual prooxidants have been investigated. Haptoglobin is a blood protein primarily synthesized by hepatocytes that is also produced locally by oligodendrocytes in the brain. Haptoglobin binds extracellular Hb, preventing Hb-mediated oxidative damage [122]. Animals that are hypohaptoglobinemic exhibit more brain damage after ICH, whereas those overexpressing haptoglobin are relatively protected. Therefore, haptoglobin is a potential therapeutic target for the prevention of brain injury following ICH [123]. Sulforaphane, a Nrf2 activator, has been shown to elevate haptoglobin and reduce brain injury in an ICH animal model [96]. Hemopexin is another blood protein known to bind heme with high affinity [124]. Hemopexin-deficient mice show increased protein oxidation, tissue heme, and augmented ICH damage [125]. This protein may also be a target to alleviate brain injury after ICH. Additional work must be performed to further establish its efficacy.

Deferoxamine mesylate (DFO), an iron chelator, is a promising agent for ICH treatment that has been confirmed to be effective in many preclinical studies [126–128]. The preliminary results in clinical trials are also encouraging. The phase I clinical trial has determined the tolerability, safety, and maximum tolerated dose of DFO in patients with ICH [129]. The phase II trial (High Dose Deferoxamine [HI-DEF] in Intracerebral Hemorrhage) is now underway, with the initial results indicating that DFO can reduce perihematoma edema, a major predictor of clinical outcome [130].

Other possible interventional targets include prooxidant enzymes, which are activated during ICH. Theoretically, inhibiting or deactivating these enzymes would be beneficial. However, controversy remains regarding the use of prooxidase inhibitors. For example, the beneficial effect of apocynin given 2 h after ICH is not achieved by its acting as an intracellular inhibitor of NADPH oxidase [71]. Tetrahydrobiopterin, which has been reported to limit the superoxide generation from NOS and chemically reduce superoxide, fails to reduce neurological deficits 24 h after ICH in mice [131]. One possible reason for this inefficacy is that many prooxidases consist of several isoforms, and their functions usually differ or can even be opposing [72, 132]. Completely, and not selectively, suppressing their activation would negate the benefits gained from some protective isoenzymes. It would be useful to identify the agent specifically acting on the detrimental isoform for a certain prooxidase.

As mentioned above, the various effects of heme oxygenases (HO-1 and HO-2) relevant to different ICH models indicate that they are challenging targets in the treatment of ICH [84, 133]. HO inhibitors may attenuate the neurotoxicity of the iron release from heme/hemin decomposition, but the toxicity of heme can enhance oxidative damage. A combination approach using two or more agents to increase HO activity while detoxifying iron with chelators has been proposed [82].

### 8.2. Scavenging Excessive ROS/RNS

Because of the impaired defense system after ICH, an alternative treatment is to neutralize the overproduced ROS and restore the normal function of endogenous antioxidant enzymes and scavengers. There is substantial support for the use of free radical scavengers in the management of brain injury secondary to ICH. Many free radical scavenging drugs have been evaluated in clinical trials to improve the outcome of ICH.

NXY-059 (disufenton sodium) is a free radical-trapping agent that significantly reduced disability and hemorrhagic transformation in acute ischemic stroke patients in the SAINT-I clinical trial [134]. Accordingly, the efficacy of NXY-059 treatment was also explored in ICH patients in the Cerebral Hematoma and NXY Treatment trial (CHANT) [135]. However, the result was disappointing, with no treatment effect observed on functional outcome, despite tolerability and safety. Edaravone is another free radical scavenger that has been marketed for clinical use in acute ischemia stroke treatment since 2001, with preclinical success in ICH [136–138]. Although good neurological function has been observed in preclinical studies, the clinical effect of edaravone in ICH remains unclear because of a lack of multicenter, randomized, double-blind clinical trials [139].

PPAR*γ* agonists have been reported to play antioxidative roles by upregulating catalase and SOD directly or activating the Nrf2 pathway, and the Safety of Pioglitazone for Hematoma Resolution in ICH (SHRINC) clinical trial has been launched [140–142]. The SHRINC study will provide important information regarding the safety and clinical outcome of PPAR*γ* agonists in ICH.

## 9. Conclusion

**Figure 2:**
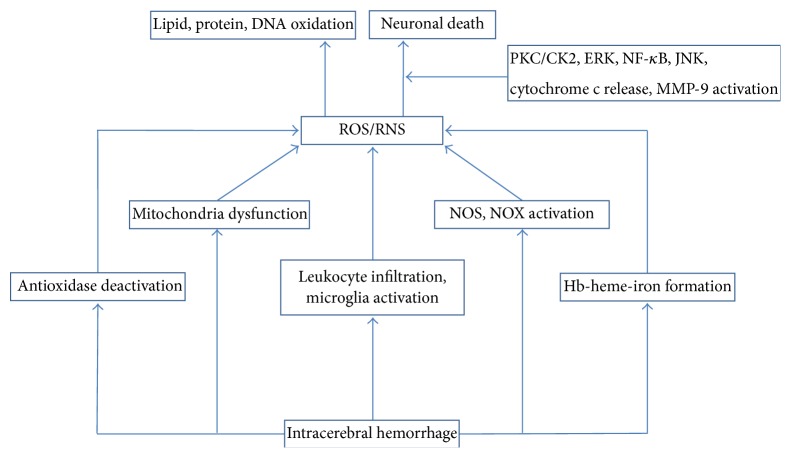
The sources of oxidative stress and the cell death pathways induced by oxidative stress following intracerebral hemorrhage. Oxidative stress after ICH is a consequence of prooxidant overproduction as well as deactivation of antioxidases such as SOD. The Hb-heme-iron metabolic axis due to erythrocyte lysis represents the major sources of ROS. Neuroinflammation evoked by ICH involves the activation of microglia and the infiltration of leukocyte which is another important contributor to the production of ROS. Activation of prooxidases including NOS and NOX during ICH also releases plenty of free radicals. Other factors which can generate ROS include mitochondria dysfunction. Oxidative stress causes cell death by direct oxidation of lipid, protein, and DNA or via induction of neuronal death mediated by PKC/CK2, ERK, NF-*κ*B, JNK signaling pathways as well as cytochrome c release, and MMP-9 activation. PKC: protein kinase C; ERK: extracellular signal-regulated kinase; NF-*κ*B: nuclear factor kappa B; JNK: c-Jun N-terminal kinase; ROS: reactive oxygen species; RNS: reactive nitrogen species; NOS: nitric oxide synthase; NOX: nicotinamide adenine dinucleotide phosphate oxidase; MMP-9: matrix metalloproteinases-9.

OS has been established as an important pathogenesis of brain injury in ICH. Upon bleeding into the parenchyma, elevated glutamate, infiltrating inflammatory cells, and the metabolic products of erythrocyte lysis are the sources of active free radical generation. Free radical overproduction is accompanied by prooxidase activation and antioxidase inhibition, causing OS in ICH. The direct biomolecule oxygenation and indirect cell death signaling pathway activations by ROS/RNS are responsible for the OS-induced brain damage after ICH (Figure 2).

Future research should focus on developing new antioxidant compounds that can both block the sources of oxidative stress in ICH and neutralize the existing overproduction of free radicals. More importantly, efforts should be made to identify the molecular mechanism underlying the effect of OS on cell death in ICH. Additionally, because ICH-induced brain damage is ascribed to a complex pathogenic mechanism, focusing on one specific pathway, such as single antioxidant treatment, is not sufficient to achieve significant clinical improvement. Therefore, one drug with multifaceted function or combined surgical and pharmaceutical treatment or two or more drug interventions with distinctive mechanisms may be promising future treatments. For these reasons, the ultimate results of the clinical trials of DFO and pioglitazone in ICH are high anticipated because both drugs have multiple beneficial effects and reduce oxidative damage [140, 143].
